# Epidemiology of Carbapenemase-Producing Enterobacteriaceae and *Acinetobacter baumannii* in Mediterranean Countries

**DOI:** 10.1155/2014/305784

**Published:** 2014-05-13

**Authors:** Nassima Djahmi, Catherine Dunyach-Remy, Alix Pantel, Mazouz Dekhil, Albert Sotto, Jean-Philippe Lavigne

**Affiliations:** ^1^National Institute of Health and Medical Research, U1047, Faculty of Medicine, Montpellier 1 University, 30908 Nîmes Cedex 02, France; ^2^Department of Microbiology, University Hospital Ibn Rochd, 23000 Annaba, Algeria; ^3^Department of Microbiology, University Hospital Caremeau, 30029 Nîmes Cedex 9, France; ^4^Departement of Infectious Diseases, University Hospital Caremeau, 30029 Nîmes Cedex 9, France

## Abstract

The emergence and global spread of carbapenemase-producing Enterobacteriaceae and *Acinetobacter baumannii* are of great concern to health services worldwide. These **β**-lactamases hydrolyse almost all **β**-lactams, are plasmid-encoded, and are easily transferable among bacterial species. They are mostly of the KPC, VIM, IMP, NDM, and OXA-48 types. Their current extensive spread worldwide in Enterobacteriaceae is an important source of concern. Infections caused by these bacteria have limited treatment options and have been associated with high mortality rates. Carbapenemase producers are mainly identified among *Klebsiella pneumoniae*, *Escherichia coli*, and *A. baumannii* and still mostly in hospital settings and rarely in the community. The Mediterranean region is of interest due to a great diversity and population mixing. The prevalence of carbapenemases is particularly high, with this area constituting one of the most important reservoirs. The types of carbapenemase vary among countries, partially depending on the population exchange relationship between the regions and the possible reservoirs of each carbapenemase. This review described the epidemiology of carbapenemases produced by enterobacteria and *A. baumannii* in this part of the world highlighting the worrisome situation and the need to screen and detect these enzymes to prevent and control their dissemination.

## 1. Introduction

Carbapenems are *β*-lactam group of drugs that are often used as antibiotics of last resort for treating infection due to multidrug-resistant Gram-negative bacilli. They are also stable even in response to extended-spectrum (ESBL) and AmpC *β*-lactamases. However, this scenario has changed with the emergence in the last few years of carbapenem resistant bacteria both in nonfermenters (*Acinetobacter baumannii* and* Pseudomonas aeruginosa*) and in fermenters (Enterobacteriaceae) Gram-negative bacilli [1].

Resistance to carbapenems is mediated mostly by two main mechanisms: (i) production of a *β*-lactamase (derepressed cephalosporinase or ESBL) with nonsignificant carbapenemase activity combined with decreased permeability due to porin loss or alteration; (ii) production of a carbapenem-hydrolyzing *β*-lactamase [2].

Carbapenemases have now become a major concern worldwide [3, 4]. They are an increasing concern for global healthcare due to their association with resistance to *β*-lactam antibiotics and to other classes of antibiotics such as aminoglycosides, fluoroquinolones, and cotrimoxazole [5]. Thus they reduce the possibility of treating infections due to multidrug-resistant strains [6]. The first description of carbapenemase-producing enterobacteria (NmcA) was in 1993 [7]. Since then, large varieties of carbapenemases have been identified belonging to three molecular classes: the Ambler class A, B, and D *β*-lactamases [8]. They have emerged and diffused in different parts of the world, including Mediterranean countries, in recent years [2–6, 9]. These enzymes are carried either on chromosome or acquired via plasmids [10].

The aim of this review is to describe the epidemiology of the main carbapenemases circulating in the Mediterranean countries, a region of the world with a great diversity and population mixing. This region includes 11 European countries (Albania, Bosnia, and Herzegovina, Croatia, Spain, France, Greece, Italia, Malta, Montenegro, Monaco and Slovenia), 5 Asian countries (Cyprus,  Israel,  Lebanon,  Syria,  Turkey) and 5 African countries (Algeria, Egypt, Libya, Morocco, Tunisia).

## 2. Class A Carbapenemases

### 2.1. Enterobacteriaceae

A variety of class A carbapenemases have been described:  some are chromosome encoded (NmcA, Sme, IMI-1, SFC-1) and others are plasmid encoded (KPC, IMI-2, GES derivatives such as GES-1, GES-2, GES-4, and GES-5) but all effectively hydrolyze carbapenems and are partially inhibited by clavulanic acid [8].

KPCs (acronym for* K. pneumoniae* carbapenemase) are the most frequently encountered enzymes in this group [2]. Since the first report of this enzyme in 1996 isolated from a clinical* Klebsiella pneumonia* strain in North Carolina, USA [11], the KPC producers had spread around the world and are becoming a major clinical and public health concern [12].

Several KPC clones are disseminating harboring different multilocus sequence type, *β*-lactamase content, and plasmids. However the *bla*_KPC_ genes are flanked by the same transposon* Tn*4401 located on conjugative plasmids and are horizontally transferred [13]. This gives to this enzyme an extraordinary spreading capacity [14]. They have been detected more often in* Klebsiella *spp. [2] but have also been reported in other Enterobacteriaceae [15]. Thirteen variants of KPC are known so far; KPC-2 and KPC-3 are the most frequent worldwide variants [16]. The mortality rate due to infection with a KPC producer ranged from 25% to 69% [2, 17].

**Figure 1:**
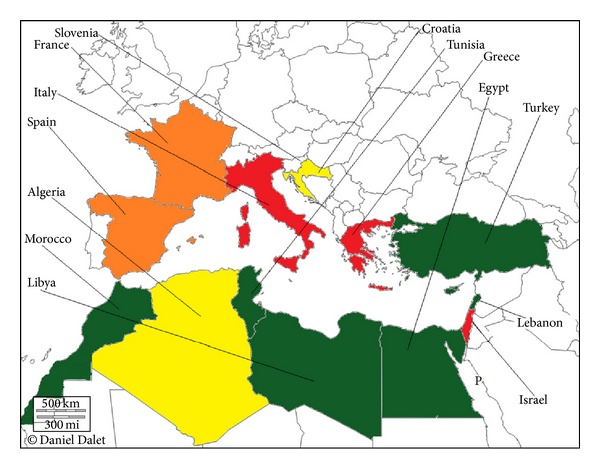
Geographic distribution of KPC enzymes in Mediterranean countries. White, no case reported; yellow, single KPC-producing isolates; green, some outbreaks of KPC-producing isolates; orange, several outbreaks of KPC-producing isolates; red, endemicity of KPC-producing isolates.

The first outbreak of KPC-producing * K. pneumoniae* outside the United States was described in Israel in 2006 [18]. This strain belonged to the pandemic clone ST258, suggesting an importation from the USA [19]. Moreover, a large range of enterobacteria producing these variants was described in Israel [20–26]. Since then, many studies have reported outbreaks of KPC producers in enterobacterial isolates in many Mediterranean countries (Figure 1), in which most cases have been reported so far in Greece, where the situation can be described as endemic [27, 28]. Moreover a recent study showed a wide dissemination of KPC-producing strains to many healthcare institutions in Italy [2, 29]. KPC producers became the most prevalent carbapenemase found in this country [30]. Spain and France have recently described a rapid increase of cases [31, 32]. Single or sporadic hospital outbreaks caused by KPCs isolated from various species were reported [32–34]. KPC-2 is clearly the most prevalent variant in Europe [12, 35]. In most of the cases reported from France, the patients had been transferred from a country where KPC enzymes are endemic (e.g., Israel, Greece, USA, or Italy) [34]. Croatia is another Mediterranean country affected [36].

To date, there is no description of class A carbapenemases from North African countries. However, KPC producers have already been isolated in an* E. coli *strain in Algeria (N. Djahmi et al., unpublished data).

### 2.2. *Acinetobacter baumannii*

Among the class A carbapenemases, KPCs and GES-type have been described in* A. baumannii* [37]. KPC-2, KPC-3, KPC-4, and KPC-10 variants were identified in 10* A. baumannii* clinical isolates collected in 2009 from 17 hospitals in Puerto Rico [38].

In Mediterranean countries, only GES-type carbapenemase was reported. A GES-14-producing* A. baumannii* clinical strain was isolated in France. This strain was demonstrated to confer resistance to all *β*-lactams, including carbapenems [39]. Very recently, an emergence of GES-11 was reported from Turkey [40]. Some strains coexpressed both OXA-23 and GES-11. They belonged to ST2, being part of the worldwide distributed clone II group.

## 3. Class B Carbapenemases

### 3.1. Enterobacteriaceae

Class B metallo-*β*-lactamases (MBLs) are mostly of the Verona integron-encoded metallo-*β*-lactamase (VIM) and IMP types and, more recently, of the New Delhi metallo-*β*-lactamases-1 (NDM-1) type [8, 41]. MBLs can hydrolyze all *β*-lactams except monobactam (e.g., aztreonam) [41]. Their activity is inhibited by EDTA but not by clavulanic acid [41].

IMP-1 was the first MBL reported in* Serratia marcescens* from Japan in 1991 [42]. Since then, MBLs have been observed worldwide [8, 41]. The most commonly found class B carbapenemases are of the VIM type [43], which has been identified in all continents [44]. The death rates associated with MBL producers are high (18% to 67%) [2, 45].

Italy was the first Mediterranean country to report acquired metallo-*β*-lactamases, with sporadic isolates of VIM-4-producing* K. pneumoniae* and* Enterobacter cloacae* [8, 46]. Since then, single or sporadic hospital outbreaks caused by VIM-1 like enzymes were described from various regions in this country [47, 48]. However, such VIM-producing Enterobacteriaceae have not undergone wide dissemination, unlike that observed in Greece during the same period [49]. Endemicity of VIM- and IMP-producing* Klebsiella pneumoniae* strains has now been noted in Greece [8, 41]. Additionally, outbreaks and single reports of VIM- or IMP-type producers have been reported in several countries of Mediterranean area, such as France [50, 51], Spain [33], Morocco [52], Egypt [53, 54], Algeria [55], and Tunisia [56].

Most recently reported, NDM-1 enzyme is spreading rapidly worldwide [44] notably Central and South America that represented the last zone without description of this enzyme [57, 58]. NDM-1 was initially identified in* E. coli *and* K. pneumoniae* in a patient returning to Sweden from India in 2008 [59]. Most of the outbreaks indicated a link with the Indian subcontinent, in some cases with the Balkan countries [60], and the Middle East [61]. Five minor variants of NDM-1 (NDM-2 to NDM-6) have been now identified in enterobacteria and very recently, a novel variant NDM-7 was detected in* E. coli* in France [62]. Contrarily to other carbapenemase genes, *bla*_NDM-1_ is not associated with a single clone. Thus NDM-1 has been identified mostly in nonclonally related* E. coli* and* K. pneumoniae* and to a lesser extent in other enterobacterial species [63]. These enzymes are encoded on highly transmissible plasmids that spread rapidly between bacteria, rather than relying on clonal proliferation. The strains harboring NDM are broadly resistant to many other drug classes in addition to *β*-lactams and carry a diversity of other resistance mechanisms, which leaves few treatment options (tigecycline or colistin) [63, 64]. NDM-1 producers have been reported in the environment and in the community [2, 63]. They have been identified in Enterobacteriaceae species around the world [59] highlighting the ability of this gene to disseminate in bacteria [65]. Moreover NDM-1 has been identified in* E. coli* ST131, a well-known source of community infections [66, 67].

**Figure 2:**
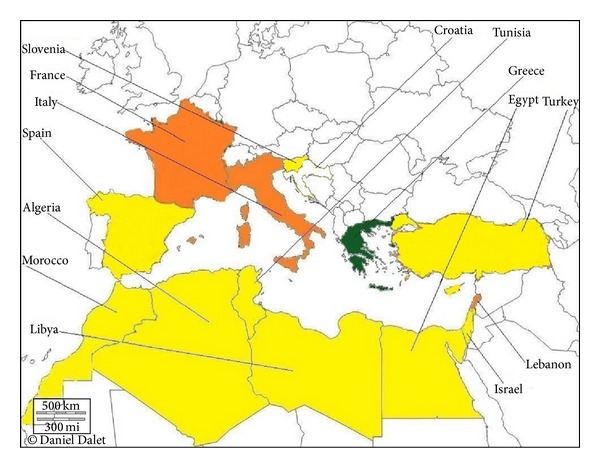
Geographic distribution of NDM type producers in Mediterranean countries. White, no case reported; yellow, sporadic NDM-producing isolates; green, emerging outbreak of NDM-producing isolates; orange, single hospital outbreaks of NDM-producing isolates.

Single or sporadic hospital outbreaks caused by NDM-1 producing enterobacterial strains were reported from many countries in Mediterranean area (Figure 2): France [68, 69], Italy [70], Lebanon [71], Morocco [52, 72], Spain [33, 73–75], Tunisia [76], and Turkey [77, 78]. Very recently, NDM-5 was identified in* E. coli* in Algeria (Sassi et al., unpublished data). There are no published data yet from Libya, but a very recent study has reported identification of NDM-1 in* K. pneumoniae* from patient transferred from Libya to Tunisia [76], indicating the emergence of this enzyme resistance in Mediterranean countries. Finally an emergence of NDM-producing* K. pneumoniae* was recently reported in Greece [79].

### 3.2. *Acinetobacter baumannii*

To date, four groups of MBLs have been identified in* A. baumannii*: IMP-like, VIM-like, SIM-like, and recently the NDMs [80].

The first MBL identified in* A. baumannii* strains was IPM-2 reported in 2000 from Italy [81]. Since then, IMP-like, VIM-like, and SIM-like have been sporadically reported in some parts of the world [82], including Mediterranean countries, especially in Greece and Italy [81–85]. Concerning NDM producers,* A. baumannii* bacteria harboring these enzymes were increasingly observed around the world [86] notably in Mediterranean countries. They were detected in North Africa: Algeria [87, 88] and Libya (isolated from a patient transferred from Libya to Denmark) [89]; in Europa: France [87, 90, 91] and Slovenia [86]; and in Turkey [92]. The isolation of an NDM-1-producing* A. baumannii* in a Czech patient repatriated in 2011 from Egypt was described [93]. In France, the emergence of imported cases of NDM-1-producing* A. baumannii* was linked with Algeria [87, 90]. The strains belonged to ST85, the main clone isolated in Mediterranean countries [90, 91]. Finally, another clone NDM variant, NDM-2, was found in* A. baumannii* isolates in Egypt [94] and Israel [95].

## 4. Class D Carbapenemases

### 4.1. Enterobacteriaceae

Class D *β*-lactamases, also named OXAs for oxacillinases include 232 enzymes with few variants, possessing the same carbapenemase activity [96]. Initially OXA *β*-lactamases were reported from* P. aeruginosa* but until now, these carbapenemases have been detected in many other Gram-negative bacteria, including Enterobacteriaceae [16].

OXA-48 represents the main enzyme isolated around the world. This enzyme hydrolyses penicillins but has a weak activity against carbapenems or extended-spectrum cephalosporins (third generation cephalosporin, aztreonam) [2]. However, its frequent association with ESBL (notably CTX-M-15 enzyme) increases the level of resistance to carbapenem. Its activity is not inhibited by EDTA or clavulanic acid [2], tazobactam, and sulbactam, whereas its activity may be inhibited by NaCl* in vitro* [96, 97]. Its high level of resistance to temocillin is interesting to detect this enzyme [98, 99]. A point mutant analog of OXA-48, namely, OXA-181, with similar carbapenemase activity, has been identified in enterobacterial strains from India [100, 101] and from patients with a link to the Indian subcontinent [100, 102]. Further analysis of the OXA-48-producing isolates demonstrated that this enzyme was not exclusively linked with a single clone, and the *bla*_OXA-48_ gene was associated with either transposon* Tn*1999 or transposon* Tn*1999.2 within transferable nontypable plasmids of 70 or 150 kb [103]. The death rates associated with OXA-producers are unknown.

**Figure 3:**
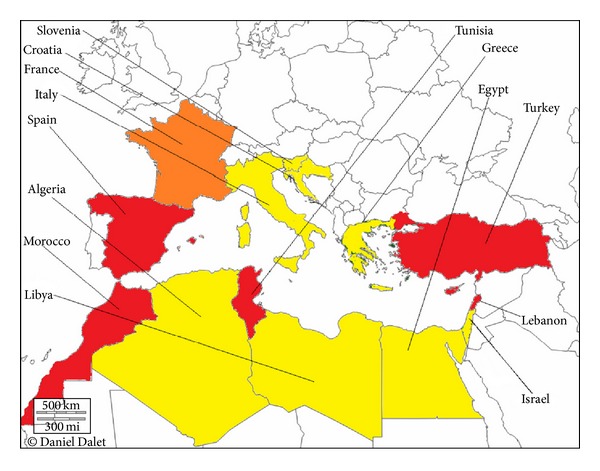
Geographic distribution of OXA-48 type producers in Mediterranean countries. White, no case reported; yellow, single OXA-48-producing isolates; orange, several outbreaks of OXA-48-producing isolates; red, nationwide distribution of OXA-48-producing isolates.

OXA-48 was initially identified in* K. pneumoniae* isolate from Turkey in 2001 [104]. Since then, OXA-48 producing strains have been extensively reported as sources of nosocomial outbreaks in many parts of the world notably in Mediterranean countries [105–110] (Figure 3): Croatia [111], Egypt [54], France [109], Greece [112], Israel [113, 114], Italy [53], Lebanon [71, 115, 116], Libya [117], Slovenia [118], Spain [33, 119], Tunisia [120], and Turkey [106]. Moreover, this enzyme disseminated in various Enterobacteriaceae species [2, 96]. To date OXA-48 represents the most common carbapenemase type circulating in this part of the world notably in Spain [33] and France [109]. The Middle East and North Africa are considered as reservoirs of OXA-48 producers [121]. In the last few years, a nosocomial dissemination of OXA-48-producing Enterobacteriaceae has been reported in different hospitals in Morocco [122]. This problem was exacerbated by the occurrence of this enzyme in community [123] and in environment [124] suggesting that OXA-48 is endemic in this country [122]. More recently, the identification of the *bla*_OXA-48_ gene in a* K. pneumoniae* isolate has been reported in Algeria (N. Djahmi, personal data).

### 4.2. *Acinetobacter baumannii*

The class D carbapenemases (oxacillinases) are by far the most prevalent carbapenemases in* A. baumannii* [125, 126]. They can be grouped into six subclasses: intrinsic chromosomal OXA-51-like, among which there are over 70 variants and the acquired OXA-23-like, OXA-24/40-like, OXA-58-like, OXA-143-like, and OXA-235-like *β*-lactamases [97, 127].

The first case of OXA-type enzyme was reported from a clinical* A. baumannii* isolate detected in Scotland in 1985. It was initially named ARI-1 (*Acinetobacter* resistant to imipenem) [128] and renamed OXA-23 after sequencing [129].* A. radioresistens* was identified as the progenitor of the *bla*_OXA23_-like gene [130].

Nosocomial outbreaks or sporadic cases caused by carbapenem-resistant* A. baumannii* producing these OXA-enzymes have been reported worldwide [80, 131–133].* A. baumannii *epidemic strains were assigned to international clonal lineages I or II [134], with recent studies reporting the spread of genetically related epidemic clone of OXA-23-producing* A. baumannii* and belonging to IC-II within the Mediterranean region [135–137]. The *bla*_OXA-23_ gene was either located on the chromosome or on plasmids and was associated with four different genetic structures, with the most frequent being transposons* Tn*2006 [134].

The emergence and spread of several outbreak or sporadic* A. baumannii* strains producing OXA-23-like enzymes have been reported around the world [134]. During a long period, the *bla*_OXA-58_ carbapenemase gene has been predominated among carbapenem-resistant* A. baumannii* isolates in various Mediterranean countries [85]. Since 2009, a replacement of *bla*_OXA-58_ gene with *bla*_OXA-23_ gene has been reported and it became the most prevalent carbapenemase-encoding gene circulating in the Mediterranean region: Algeria [88, 136], Croatia [111], Egypt [138], France [139], Greece [140], Italy [135, 141], Israel [132], Spain [137, 142], Tunisia [143], and Turkey [83, 144]. The replacement of OXA-58 by OXA-23 might be explained by the selective advantage associated with the higher carbapenemase activity of OXA-23 [37, 142] and/or acquisition of carbapenem resistance through horizontal gene transfer [37].

Concerning other OXA-producers, outbreaks of OXA-72-producing* A. baumannii* were described in Croatia [145] and OXA-69 or OXA-97 in Tunisia [146, 147].

## 5. Conclusion

In recent years the emergence of carbapenem-resistant Gram-negative bacilli in Mediterranean region is an alarming problem. This part of the world is the cradle of western civilization representing nearly 475 million inhabitants (6.3% of world population). It is the location of a large population mixing explaining the importance of the dissemination of carbapenemase producers. This situation imposes a series of measures as soon as possible. These need the over-the-counter sale of indistinctly antibiotics, improving basic and extended knowledge on hygiene, the reinforcement of infection control measures, and the early and accurate detection, with restriction of the usage of carbapenems, to control the spread of these multidrug resistant organisms.
